# Personal experience and awareness of opioid overdose occurrence among peers and willingness to administer naloxone in South Africa: findings from a three-city pilot survey of homeless people who use drugs

**DOI:** 10.1186/s12954-021-00561-w

**Published:** 2022-02-11

**Authors:** Michael Wilson, Amanda Brumwell, M. J. Stowe, Shaun Shelly, Andrew Scheibe

**Affiliations:** 1 Advance Access and Delivery South Africa, 112 Stamfordhill Road, Greyville, Durban, 4001 South Africa; 2 Department of Health Behavior, University of North Carolina Gillings School of Global Public Health, Chapel Hill, NC USA; 3 Advance Access and Delivery Inc., Durham, NC USA; 4 Department of Family Medicine, School of Medicine, Faculty of Health Sciences, University of Pretoria, Pretoria, South Africa; 5 South African Network of People Who Use Drugs (SANPUD), Cape Town, South Africa; 6 TB HIV Care, Cape Town, South Africa; 7 Urban Futures Centre, Faculty of Engineering, Durban University of Technology, Durban, South Africa

**Keywords:** Drug overdose, Naloxone, Opioids, People who inject drugs, Drug-related harms

## Abstract

**Background:**

Drug overdoses occur when the amount of drug or combination of drugs consumed is toxic and negatively affects physiological functioning. Opioid overdoses are responsible for the majority of overdose deaths worldwide. Naloxone is a safe, fast-acting opioid antagonist that can reverse an opioid overdose, and as such, it should be a critical component of community-based responses to opioid overdose. However, the burden of drug overdose deaths remains unquantified in South Africa, and both knowledge about and access to naloxone is generally poor. The objective of this study was to describe the experiences of overdose, knowledge of responses to overdose events, and willingness to call emergency medical services in response to overdose among people who use drugs in Cape Town, Durban, and Pretoria (South Africa).

**Methods:**

We used convenience sampling to select people who use drugs accessing harm reduction services for this cross-sectional survey from March to July 2019. Participants completed an interviewer-administered survey, assessing selected socio-demographic characteristics, experiences of overdose among respondents and their peers, knowledge about naloxone and comfort in different overdose responses. Data, collected on paper-based tools, were analysed using descriptive statistics and categorised by city.

**Results:**

Sixty-six participants participated in the study. The median age was 31, and most (77%) of the respondents were male. Forty-one per cent of the respondents were homeless. Heroin was the most commonly used drug (79%), and 82% of participants used drugs daily. Overall, 38% (25/66) reported overdosing in the past year. Most (76%, 50/66) knew at least one person who had ever experienced an overdose, and a total of 106 overdose events in peers were reported. Most participants (64%, 42/66) had not heard of naloxone, but once described to them, 73% (48/66) felt comfortable to carry it. More than two-thirds (68%, 45/66) felt they would phone for medical assistance if they witnessed an overdose.

**Conclusion:**

Drug overdose was common among participants in these cities. Without interventions, high overdose-related morbidity and mortality is likely to occur in these contexts. Increased awareness of actions to undertake in response to an overdose (calling for medical assistance, using naloxone) and access to naloxone are urgently required in these cities. Additional data are needed to better understand the nature of overdose in South Africa to inform policy and responses.

## Background

Globally, there are an estimated 15 million people who inject drugs (PWID) [1], with 20.5% having experienced a non-fatal drug overdose event in the last 12 months. Drug overdose is responsible for substantial mortality among people who use drugs, with an estimated 109,500 people dying from opioid overdose in 2017 [2]. However, weak vital registration systems and limited surveillance systems limit the understanding of the prevalence and consequences of opioid overdose [3].

Naloxone is a semisynthetic competitive opioid antagonist with a high affinity for the μ opioid receptor and can reverse an opioid overdose. Further, naloxone acts within seconds and has a short duration of effect; it has an elimination half-life of 60–90 min [4].

The World Health Organisation (WHO) recommends naloxone for the emergency treatment of known or suspected opioid overdose with respiratory or central nervous system depression [5] and recommends naloxone distribution programmes in community settings as they reduce deaths related to opioid overdose and save costs [5]. Overdose education and training of people at risk of opioid overdose and among those likely to come into contact with people experiencing an overdose is the foundation for naloxone distribution programmes [5]. The administration of naloxone by people who are likely to be present at an overdose (e.g. peers, police, outreach workers) who could respond before medical professionals arrive increases the chances of survival [6].

### South African context

The use of drugs (other than cannabis) is illegal in South Africa [7]. There is limited data on the prevalence of heroin use and overdose. Most of the country's estimated 75,000 PWID [8] use heroin (known locally as nayope, whoonga, unga, sugars and thai) [9]. Among whom, many are homeless [10]. Further, evidence shows that the use of heroin and the prevalence of injecting drug use is on the rise [11], increasing the risk of opioid-related overdoses.

The accuracy of South Africa’s vital registration system is negatively affected by data limitations, including missing data on cause of death [12]. Opioid and other drug-related deaths are categorised under accidental poisoning by and exposure to a noxious substance [12]. The latest published data (2017) reflect 720 deaths due to accidental poisoning which equates to 1.4% of deaths due to external causes of accidental injury [12]. Overdose is not routine monitored in the drug-related surveillance collected from drug treatment and harm reduction services via the South African Community Epidemiology Network on Drug Use [11].

Naloxone is registered and available as a hydrochloride solution in an ampule, typically 0.4 mg per 1 ml injection. South Africa's Essential Medicines List includes naloxone for the management of opioid poisoning across all levels of care [13]. Harm reduction services operate in several cities, with opioid substitution therapy programmes operational in four cities, and needle and syringe services available in an additional five cities [14]. To date, no community-based naloxone distribution programmes have taken place [15].

We performed a pilot study among people who access harm reduction services in three South African cities to provide initial insights into: (1) occurrence of opioid overdose among study participants and their peers, (2) levels of comfort in calling for emergency medical assistance in the case of an overdose, (3) knowledge of how to respond to an opioid overdose and (4) comfort in carrying naloxone. The study intended to provide preliminary data on overdose and to inform future research.

## Methods

We conducted a cross-sectional survey in Durban, Cape Town, and Pretoria between March-July 2019.

### Study settings

The three cities were selected based on the availability of harm reduction services provided as well as the need identified by service providers for insight into frequency of drug overdose. At the time of the survey, outreach programmes took services to homeless people who use drugs. The teams distributed sterile injecting equipment (except in Durban where a municipal ban on the service was in place between May 2018 and June 2020), provided advice on harm reduction and safer injecting, and made referrals for psychosocial services. Opioid substitution therapy was available to a group of ± 30 people who injected opioids in Cape Town and ± 1000 people who use opioids in Pretoria.

### Eligibility criteria and sampling

We invited individuals, aged 18 years and above, who used drugs and accessed the harm reduction service in the respective city to participate. Participants were conveniently sampled. Participants were approached by outreach team members during routine outreach visits and asked to participate.

### Measures

The survey tool gathered data on the following areas: (1) socio-demographics (age, gender, race, housing), (2) drug use history (current, past, duration, and frequency of use), (3) personal history of drug-related overdose in the past year (frequency, location, drugs used, presence of others and assistance received), (4) awareness of opioid overdose among peers, (5) awareness of and likelihood of using naloxone, and (6) likely action taken in response to an overdose situation.

An opioid overdose was described to participants including the most likely signs and symptoms (slowed breathing, slurred speech, changes in heart rate, unresponsiveness, and blue lips and fingertips). Participants were instructed to report overdoses in which opioids were known or thought to be the cause. Since opioid overdose can be the result of a multiple substances, participants were able to list non-opioid drugs that were thought to be involved in the reported overdose.

Regarding “likelihood of using naloxone”, participants were given a brief summary explaining the role of naloxone in reversing an opioid overdose. It was explained to participants that in South Africa, naloxone is primarily used by emergency medical personnel to reverse an opioid overdose. Participants were asked whether they would be interested in carrying and administering naloxone in the event of an opioid overdose should future policies permit peers access to naloxone. Participants could answer “Yes”, “No”, or “Not sure”.

Regarding “likely action taken”, participants were asked what they would do to respond to a suspected opioid overdose where they were present. This question was open-ended, allowing respondents to share their response with the surveyor.

### Data collection

The study was integrated into routine outreach activities and trained outreach team members from TB HIV CARE and COSUP administered the tool in the field. The survey was administered in English and took 15–20 min to complete which included providing information about the study and obtaining verbal consent to participate.

### Data analysis

Data from the paper-based survey tool were entered manually into a Microsoft Excel database and analysed using descriptive statistics (frequencies and proportions). Data were stratified by city.

## Results

A total of 66 people participated in the study (29 in Durban, 21 in Cape Town and 16 in Pretoria). Overall, data were missing for 18% (243/1314) of responses to survey questions (18% [58/327] for socio-demographics, 31% [81/264] for drug history, 17% [36/206] for personal history of overdose, 11% [13/121] of overdose among peers, 11% [14/132] of likelihood of using naloxone and 16% [41/264] on likely action in response to an overdose).

The median age of participants was 31 years old [Interquartile range (IQR) 28–35], and the majority of participants identified as male (77%, 51/66). The majority of participants (74%, 49/66) declined to provide racial or ethnic identity. The majority of survey participants (59%, 39/66) were homeless or living in a shelter at the time of the survey. Fewer people in Pretoria reported being homeless (33%), compared to 72% of those in Durban and 56% of those in Pretoria. The most frequently used substances were heroin (79%, 52/66), cocaine (26% 17/66) and methamphetamine (23%, 15/66). Among participants, 82% (54/66) used at least daily, 38% (25/66) mixed substances in the past year, and 76% of those providing a response (25/33) had used drugs for more than five years. Cape Town-based respondents most frequently reported mixing drugs in the past year (71%), compared to 14% of Durban respondents and 38% of Pretoria respondents; however, the majority of Durban respondents (76%) did not provide a response regarding mixing drugs (Table 1).

**Table 1:** Respondent demographics and reported substance use behaviours (*n* = 66)

Respondent demographics	Total responses across all cities, *n* = 66 (%)	Durban, *n* = 29 (%)	Cape Town, *n* = 21 (%)	Pretoria, *n* = 16 (%)
*City*				
Durban	29 (44%)			
Cape Town	21 (32%)			
Pretoria	16 (24%)			
*Gender*				
Male	51 (77%)	23 (79%)	14 (67%)	14 (88%)
Female	10 (15%)	6 (21%)	4 (19%)	2 (13%)
Other/no response	5 (8%)	0 (0)	3 (14%)	0 (0)
*Race*				
Black	9 (14%)	1 (3%)	0 (0)	8 (50%)
White	7 (11%)	1 (3%)	0 (0)	6 (38%)
Mixed ancestry	1 (2%)	0 (0)	0 (0)	1 (6%)
Missing	49 (74%)	27 (93%)	21 (100%)	1 (6%)
*Age (years)*				
Median, IQ Range	31 (28.25–35)	28 (26–31)	33 (32–36)	33 (30–37.5)
Homeless	27 (41%)	21 (72%)	7 (33%)	9 (56%)
Shelter	12 (18%)	8 (28%)	0 (0)	2 (13%)
Family	6 (9%)	0 (0)	0 (0)	2 (13%)
*Current residence*				
Friends	8 (12%)	0 (0)	3 (14%)	1 (6%)
Home	9 (14%)	0 (0)	8 (38%)	1 (6%)
No response	4 (6%)	0 (0)	3 (14%)	1 (6%)
*Time using drugs*				
5 or fewer years	8 (12%)	2 (7%)	5 (24%)	1 (6%)
More than 5 years	25 (38%)	2 (7%)	11 (52%)	12 (75%)
No response	33 (50%)	25 (86%)	5 (24%)	3 (19%)
Heroin	52 (79%)	20 (69%)	17 (81%)	15 (94%)
Cocaine	17 (26%)	6 (21%)	4 (19%)	7 (44%)
*Drugs used in the past year*^a^				
Methamphetamine	15 (23%)	1 (3%)	13 (62%)	1 (6%)
Cannabis	5 (8%)	3 (10%)	0 (0)	2 (13%)
Benzodiazepine	4 (6%)	4 (14%)	0 (0)	0 (0)
Methaqualone	6 (9%)	1 (3%)	5 (24%)	0 (0)
Ecstasy	1 (2%)	0 (0)	1 (5%)	0 (0)
*Frequency of use*				
Daily	54 (82%)	21 (72%)	17 (81%)	16 (100%)
Weekly	1 (2%)	0 (0)	1 (5%)	0 (0)
Monthly	1 (2%)	1 (3%)	0 (0)	0 (0)
No response	10 (15%)	7 (24%)	3 (14%)	0 (0)
*Mixed drugs in the past year?*				
Yes	25 (38%)	4 (14%)	15 (71%)	6 (38%)
No	15 (23%)	3 (10%)	2 (10%)	10 (63%)
No response	26 (39%)	22 (76%)	4 (19%)	0 (0)
*Overdosed in past year*				
Yes	25 (38%)	12 (41%)	3 (14%)	10 (63%)
No	38 (58%)	17 (59%)	15 (71%)	6 (38%)
Not sure/no response	3 (5%)	0 (0)	3 (14%)	0 (0)

^a^People could respond to more than one option

Overdose experiences are presented in Table 2. Over one third of participants (38%, 25/66) had experienced an overdose in the past year. Of those, participants reported a median of one overdose in the past year (IQR 1–2). The largest proportion of participants reporting an overdose in the past year were from Pretoria (63%, 10/16), and the smallest proportion was from Cape Town (14%, 3/21). Almost half (48%, 12/25) of those who had experienced an overdose were "outside" when it occurred. Most (83%, 15/18) of those reporting the type of drug-related to the overdose reported using heroin, and two others reported using a heroin mixture. Almost a third (28%, 7/25) of participants did not report the kind of drug used before the overdose event. Nearly two-thirds of participants who experienced an overdose were with someone at the time of their overdose; however, 72% (18/25) did not receive help during their overdose.

**Table 2:** Reported overdose experiences by survey respondents (*n* = 66)

Overdose experience		Responses, *n* = 25	Durban (*n* = 12)	Cape Town (*n* = 3)	Pretoria (*n* = 10)
How many times have you overdosed in past year?	Median, IQ range	1 (1–2)	2 (1–3)	2 (1.5–2)	1 (1–1)
Where were you when you overdosed?	Outside	12 (48%)	9 (75%)	0 (0)	3 (30%)
	At home	2 (8%)	1 (8%)	0 (0)	1 (10%)
	At friend's house	1(4%)	1 (8%)	0 (0)	0 (0)
	At shelter	1 (4%)	1 (8%)	0 (0)	0 (0)
	No response	9 (36%)	0 (0)	3 (100%)	6 (60%)
Overdosed with what kind of drug?	Heroin	15 (60%)	6 (50%)	0 (0)	9 (90%)
	Mixture: Heroin and methamphetamine	2 (8%)	0 (0%)	2 (67%)	0 (0)
	Mixture: methamphetamine and cocaine	1 (4%)	0 (0)	1 (33%)	0 (0)
	No response	7 (28%)	6 (50%)	0 (0)	1 (10%)
Were you with someone when you overdosed?	Yes	16 (64%)	9 (75%)	0 (0)	7 (70%)
	No	8 (32%)	2 (17%)	3 (100%)	3 (30%)
	No response	1 (4%)	1 (8%)	0 (0)	0 (0)
Did help come when you overdosed?	Yes	5 (20%)	3 (25%)	0 (0)	2 (20%)
	No	18 (72%)	9 (75%)	1 (33%)	8 (80%)
	No response	2 (8%)	0 (0)	2 (67%)	0 (0)

Seventy-six per cent (50/66) of participants knew at least one person who had experienced an overdose in the past year. These participants reported a total of 106 overdose experiences among their peers. The distribution of these reported overdoses was similar across the three survey locations, with 46 (43%), 31(29%), and 29 (27%) in Durban, Cape Town, and Pretoria, respectively.

Table 3 provides knowledge around and responses to overdose. The majority of participants (64%, 42/66) had not heard of naloxone, but, 73% (48/66) indicated that they would carry naloxone after being informed about what it is. Pretoria had the highest proportion of respondents having heard of naloxone previously (56%), whereas Durban had the lowest proportion (14%). Forty per cent (10/25) of participants who overdosed in the past year had heard of naloxone. Similarly, 76% of all participants (50/66) knew at least one person who overdosed in the past year.

**Table 3:** Reported overdoses among contacts of respondents and methods of responding to overdose experiences (*n* = 66)

Knowledge of overdoses and responding to overdoses	All Responses, *n* = 66 (%)	Durban (*n* = 29)	Cape Town (*n* = 21)	Pretoria (*n* = 16)
*Know others who have overdosed?*				
Yes	50 (76%)	23 (79%)	15 (71%)	12 (75%)
No	11 (17%)	3 (10%)	4 (19%)	4 (25%)
No response	5 (8%)	3 (10%)	2 (10%)	0 (0)
*Have you heard of naloxone?*				
Yes	18 (27%)	5 (17%)	4 (19%)	9 (56%)
No	42 (64%)	20 (69%)	15 (71%)	7 (44%)
No response	6 (9%)	4 (14%)	2 (10%)	0 (0)
*Likely to carry naloxone?*				
Yes	48 (73%)	23 (79%)	9 (43%)	16 (100%)
No	6 (9%)	2 (7%)	4 (19%)	0 (0)
Not sure/did not answer	12 (18%)	4 (14%)	8 (38%)	0 (0)
*What would you do in the event of an overdose? (only one answer provided by participants)*				
Phone for medical assistance	45 (68%)	16 (55%)	17 (81%)	12 (75%)
Help	13 (20%)	7 (24%)	2 (10%)	4 (25%)
Nothing	0 (0)	0 (0)	0 (0)	0 (0)
I don't know/did not answer	8 (12%)	6 (21%)	2 (10%)	0 (0)
*Are you comfortable calling for help in the event of an overdose?*				
Yes	49 (74%)	17 (59%)	18 (86%)	14 (88%)
No	4 (6%)	4 (14%)	0 (0)	2 (13%)
Not sure/did not answer	13 (20%)	8 (28%)	3 (14%)	0 (0)
*If you are comfortable calling for help, what kind of help would you call?*				
First responder, paramedic, police	37 (56%)	18 (62%)	14 (67%)	5 (31%)
Naloxone	4 (6%)	0 (0)	4 (19%)	0 (0)
Relative, family, or friend	2 (3%)	2 (7%)	0 (0)	0 (0)
Administer help on your own	1 (2%)	1 (3%)	0 (0)	0 (0)
Not sure/did not answer	22 (33%)	8 (28%)	3 (14%)	11 (69%)
*Do you think that help would come?*				
Yes	49 (74%)	21 (72%)	13 (62%)	15 (94%)
No	4 (6%)	3 (10%)	1 (5%)	0 (0)
Not sure/did not answer	13 (20%)	5 (17%)	7 (33%)	1 (6%)

Regarding responses to overdose events, more than two-thirds (68%, 45/66) would phone for medical assistance and a fifth (13/66) would administer help to someone experiencing an overdose. The remainder (12%, 8/66) either did not respond to the question or did not know what they would do in the event of an overdose. Similarly, the majority of participants (74%, 49/66) were comfortable calling for help in the event of an overdose. Over half (56%, 37/66) would call for professional medical assistance in the event of an overdose.

## Discussion

We present, to our knowledge, the first published data of drug overdose among people who use drugs accessing harm reduction services in selected cities in South Africa. The findings from this small pilot study highlight the likely burden of overdose. The study points to probable significant (and largely undocumented) overdose-related morbidity and mortality among marginalised people who use drugs in South Africa. This initial study highlights the critical need to better quantify and understand overdoses to inform policy and programming. The characteristics of this sample are similar to people who access harm reduction services at organisations that are part of the South Africa Community Epidemiology Network on Drug Use [14].

This study found that opioid overdoses are occurring within the population of people who use drugs in the selected cities. Over a third of the participants experienced an overdose in the last year. Over two-thirds were aware of overdoses occurring among their peers, presenting an important opportunity to train peers in harm reduction principles, including recognising and responding to an opioid overdose. Our pilot study has also demonstrated the willingness of people who use drugs who access harm reduction services to report personal experiences of drug overdose. The ability of this approach to obtain information on overdose experiences among peers suggests that the community of people who use drugs share their experiences among themselves. The sense of community is important because peers are often the first ones that can phone for medical assistance in the case of an overdose. In many settings globally, peers play a critical role in saving lives by reversing potentially lethal overdoses [16].

Several known risk factors associated with fatal drug overdose were reported. These risk factors include homelessness, daily injection of heroin and polysubstance use [17]. This information indicates a significant opportunity to direct increased efforts to reduce the risk of morbidity and mortality among this group of individuals. Further risk reduction can also be achieved through increased access to opioid substitution maintenance therapy [5]. Further, an overdose can be mitigated through community awareness and training, and the wide distribution of naloxone [5].

While the sample is small and comprised mostly of homeless people, there was heterogeneity in the responses. Caution should be exercised in generalising from these participants. However, despite the small sample, the majority (64%) had never heard of naloxone, yet most respondents were willing to carry it on their person in case of an overdose, should it become available and accessible. Given that most participants who had overdosed in the past year reported that they were not alone at the time of the overdose, participants' willingness to carry naloxone presents an opportunity to equip the people closest to those at risk of overdose with a tool proven to prevent death. The findings align with the WHO’s guidelines for community management of opioid overdose [5]. The WHO guidelines recommend that people who are likely to witness an opioid overdose, very often friends and family members of people who use opioids, should be given access to naloxone and training on how to recognise and respond to an overdose [5]. As a result, at least 15 countries globally have implemented programmes which include access to naloxone and overdose training, demonstrating an increase in knowledge and competencies around responding to an opioid overdose [18, 19]. The rapid scale-up of overdose prevention programmes (including take home naloxone) is possible in low- and middle-income settings, provided there is political will [20]. Programmes that improve peer and family responses to overdose situations decrease rates of overdose deaths when compared to programmes that do not include family and peers [21]. Brief education and training of opioid users in recognising and responding to an overdose of a peer improved overdose response [22].

The existing legislation in South Africa does not include Good Samaritan laws [23]. The provision of naloxone is limited to self-use only, requiring a prescription by a medical doctor. The risk of prosecution makes it challenging to ensure peers, who are often opioid users themselves are equipped to respond to a potentially lethal overdose. Neither the draft national standards for emergency medical services (2021) [24] nor the Health Professions Council of South Africa’s clinical practice guidelines for emergency service providers (2018) [25] include a requirement for emergency medical service providers to report drug overdoses to the police.

Finally, the study found that most people were comfortable calling for help in the event of an overdose. However, a notable proportion (26%) were either uncomfortable calling for help or did not know whom to call. Globally, there is a reluctance, particularly among people who use drugs, to calling for help [26]. One of the most significant predictors of calling for help is the drug policy environment and the related charges one might face if arrested at the site of an overdose [27]. There is very little data on the facilitators and barriers to calling for help to respond to an overdose in South Africa. A report by the South African Network of People Who Use Drugs revealed that people who use drugs experienced discrimination and delayed response times when calling ambulances to respond to medical emergencies [28]. In the USA, people most often cite the fear of police involvement as the reason for low rates of calling for help in the case of a drug overdose [26, 29]. Good Samaritan Laws provide limited indemnity from prosecution if someone responds to an overdose and calls 911 to seek medical help. In the USA, Washington was the second state to implement Good Samaritan laws. In a survey of 355 opiate users in Washington, when informed of the laws, 88% reported they would phone for medical assistance in the case of an opioid overdose [30].

In South Africa, the hostile engagement between people who use drugs and law enforcement continues, as noted by harassment and often confiscation and destruction of injecting equipment (SAMRC, 2020). Encouragingly, efforts are ongoing to enable collaboration between health and security actors towards public health and safety [31].

Over the past decade, as global harm reduction efforts have focused on making naloxone more accessible and available in overdose situations, first responders have been equipped with naloxone [32]. In many settings globally, law enforcement officers are frequently first to arrive on the scene of an emergency. When officers carry and are prepared to use, they can administer naloxone before other responders arrive, increasing the likelihood of effective overdose reversal [32].

In the case of an opioid overdose, death does not usually occur immediately, often allowing time for a life-saving intervention. Overall, 76% of individuals surveyed in this study were aware of opioid overdoses occurring among their peers; however, very few people who experienced an opioid overdose received medical attention; hence, it is critical to ensure that those who are most likely to be at the scene of a drug overdose have the capacity to recognise and respond to an opioid overdose. Self-report data from PWID are critical to understanding personal and witnessed overdose events. Many cities around the world have begun training bystanders and peers on overdose prevention and response, including self-reporting.

Peers and bystanders represent a critical part of any comprehensive overdose response plan. For example, the WHO and the United Nations Office on Drugs and Crime’s *Stop Overdose Safely* project in Kazakhstan, Kyrgyzstan, Tajikistan and Ukraine demonstrated the effectiveness of community naloxone distribution [20]. The project involved the training of 14,263 potential opioid overdose witnesses over eight months. Trainees were provided with take home naloxone. Thirty-five per cent (478/1388) of participants engaged in the project evaluation cohort witnessed an overdose within six months following the training, among whom 89% used naloxone with 98% of the victims surviving [20]. There is an important need to maximise the potential role of bystanders and communities of people who use opioids in South Africa and other African contexts to respond to opioid overdoses.

Our South African pilot study has several limitations. First, the small sample and convenience sampling of participants who access harm reduction services limit the generalisability of findings. The available resources, accessibility of participants, and feasibility of integrating the survey into service delivery influenced the sample size. However, the pilot study has established preliminary data that points towards a high prevalence of overdose among people who use drugs. The value of assessing the feasibility and acceptability of integrating overdose assessment into harm reduction service in a more robust manner has been demonstrated. The small size of this study limits the degree to which it can be used to directly inform policy.

Second, the survey tool was not validated, and the amount of missing data limited the analysis that was possible. Missing data fields were caused primarily by the use of paper-based tools with free text and the option of participants to decline answering questions. Notably, many participants declined to provide demographic information or information about their experiences with overdose.

Third, the study did not explicitly ask participants about access to or use of health-related or harm reduction services. However, given that recruitment was performed by harm reduction service providers, it is possible that the study cohort likely has increased exposure to information about responding to an overdose. As a result, it is likely that they would have felt more comfortable calling for help or administering aid when responding to an overdose. It cannot be assumed that the same level of knowledge and willingness to phone for medical assistance would be reported among groups with less exposure to harm reduction organisations.

## Conclusion

Our pilot study demonstrates that there is likely to be a high prevalence of overdose among people who use drugs in South Africa, particularly among people who inject opioids. Opioid overdose is likely contributing to notable morbidity and mortality among people who inject opioids in South Africa. The study also highlights that people who use drugs are willing to respond to overdoses with naloxone and to call for help. The findings suggest that there is a need to strengthen efforts to raise awareness about overdose responses, including around naloxone. Notably, the study highlights the need to move towards naloxone being available for use by people likely to witness an overdose. Further data are needed to quantify and understand the context and substances involved in overdoses. Findings from future research and surveillance can inform advocacy efforts towards changing policy and programming. Policy changes include the rescheduling of naloxone to enable purchase without a prescription and administration by trained people likely to come across people experiencing an opioid overdose. Future programmatic changes could include community distribution of naloxone through harm reduction and social service providers and the police. All policy and programme changes should protect the responders and overdose victims from drug-related arrest and criminal sanctions. Until more robust data are gathered, the findings from our pilot demonstrate the need within harm reduction service providers to raise awareness of the risks associated with a drug overdose, how to respond in the event of a drug overdose, and to advocate for increased community access to naloxone for peers and first responders.

## Data Availability

Data generated from this study can be accessed on request to the authors.

## Abbreviations

COSUP: Community Oriented Substance Use Programme
PWID: People who inject drugs
SACENDU: South African Community Epidemiology Network on Drug Use
SANPUD: South African Network of People Who Use Drugs
WHO: World Health Organisation
